# “Responsible” or “Strange?” Differences in Face Mask Attitudes and Use Between Chinese and Non-East Asian Canadians During COVID-19’s First Wave

**DOI:** 10.3389/fpsyg.2022.853830

**Published:** 2022-03-17

**Authors:** Ying Shan Doris Zhang, Kimberly A. Noels, Heather Young-Leslie, Nigel Mantou Lou

**Affiliations:** ^1^Department of Psychology, University of Alberta, Edmonton, AB, Canada; ^2^Department of Psychology, University of Victoria, Victoria, BC, Canada

**Keywords:** COVID-19, face mask, interethnic variations, attitude, public health, well-being

## Abstract

Early in the COVID-19 pandemic, journalists and scholars noted differences between Asians and North Americans in their support for public mask use. These differences were primarily assumed to be due d to variations in ethnocultural norms and practices. To better ascertain people’s motives for wearing masks and potential cultural differences in these rationales, this comparative, mixed-methods research examines Chinese and non-East Asian Canadians’ mask use attitudes utilizing online group interviews (Study 1) and a nation-wide survey (Study 2) Study 1, conducted in the early stages of the pandemic, captured an ambivalent, yet evolving attitude toward public mask use among the non-East Asian Canadians, which differed from their Chinese counterparts who more uniformly perceived mask use favorably. Study 2, conducted 2 months later, suggests that both groups primarily wore masks for disease protection- and prevention-related reasons. However, age and education appeared to influence the mask wearing frequency of the non-East Asian Canadians, for whom public mask use was less prevalent and normative. The attitudinal differences in public mask use call for targeted strategies to support mask wearing for different ethnocultural groups, which may be achieved partially through enhancing interethnic understanding on the diversified use of and opinions about masks. The findings suggest that favorable social norms, along with evidence-based information campaigns involving personal appeals may encourage greater mask use by the non-East Asian population.

## Introduction

Face masks have become a daily necessity during the COVID-19 pandemic. Because the SARS-CoV-2 virus is transmitted through respiratory droplets and can be airborne (Government of Canada, 2021a), mask wearing is one of the main preventative health measures to stop its spread (Kaslow et al., 2020; Howard et al., 2021). Public mask use, however, has not always been well received in North America. The rise of various mask mandates to help contain the spread of COVID-19 was perceived by angry anti-maskers as restrictions that infringe on their rights to freedom and democracy, and more extremely, as a way for the government to exercise control over its people (Warick, 2020). As a result, anti-mask rallies spread across North America, and the public became further divided in regards to support for public mask use in the pandemic (Bogart, 2020).

Such incidents mark a stark contrast to many East Asian countries, where people are seemingly more willing to wear masks in public areas to collectively combat COVID-19 (Greenhalgh et al., 2020; Zhao and Knobel, 2021). This cross-national difference is reflected in interethnic differences in Canada and the United States, where some East Asian residents voluntarily began to wear masks in public areas for personal protection prior to public health officials’ recommendations (He et al., 2020). This difference in cultural practices around public mask wearing could provide insight into how to develop mask-wearing practices among North Americans. Such knowledge might also ultimately help to mitigate xenophobia against East Asian mask wearers in North America (Cheung et al., 2020; Lou et al., 2021).

To explain the differences in public mask use between different ethnocultural groups, existing public health and social psychology literature delved into the impact of the nations’ previous experiences with epidemics and environmental pollutants (Song et al., 2020); differences in perceived threat across minority and majority groups (Niño et al., 2021; Reiter and Katz, 2021); and social norms for mask use (Young and Goldstein, 2021). Although social norms are widely argued to be important, relatively little empirical research has examined people’s attitudes toward public mask use. These attitudes might reasonably be seen to contribute to the observed normative differences across ethnocultural groups. Moreover, they can be expected to predict people’s behavior around public mask use.

To further our understanding on the divergent support for masks, this research examines the personal attitudes of Chinese and non-East Asian Canadians around public mask use, and compares their thoughts, feelings, and mask-wearing behaviors prior to and during the COVID-19 pandemic. We also examine how different attitudes predict mask-wearing across these two groups. To this end, we first review the literature on interethnic differences in mask use, and describe its implications for public health and interethnic relations, before proceeding to the current studies.

## National Variations in Mask Use

In East Asia, public mask use is both a descriptive and a prescriptive norm, which refers to “the frequency with which given behaviors occur,” and “moral values and societal standards about behaviors,” respectively (Brauer and Chaurand, 2010, p. 491). Specifically, mask wearing is a frequent practice in most East Asian countries (e.g., China, Japan, South Korea; Chan, 2020) across different contexts, for a variety of reasons. For instance, aside from containing the spread of respiratory diseases, masks are also used to block skin exposure to strong UV rays and/or cold air, prevent breathing in irritants (e.g., pollens and pollutants), and create anonymity in public contexts (Flaskerud, 2020). In several East Asian countries, masks have evolved into beauty and fashion accessories that can be coordinated with clothing choices (Egli, 2015; Miyazaki and Kawahara, 2016). Scholars have associated the widespread use of masks in East Asian countries with a long-standing history of national responses to previous epidemics, along with formalized policies and laws requiring mask use in public domains (Yang, 2014; Greenhalgh et al., 2020). Further, the prescriptive norm for mask use may have been strengthened by the collective cultural norms of many East Asian societies, which prioritize civic responsibility and community well-being in critical public health crises (Chan, 2020).

Unlike East Asia, mask use was less pervasive in Western societies prior to the COVID-19 pandemic (Lai et al., 2021). Particularly in North America, masks were predominately used for medically related reasons (O’Kane and Baldwin, 2020). Although mask use in public settings was compulsory in Canada during the 1918–1919 influenza (Spanish Flu; Goldernberg, 2018), it ceased as a common practice after that pandemic had passed. There is no definitive explanation as to why, in contrast with East Asian societies, public mask use was discontinued in North America (Chan, 2020). One possible explanation might be that the relatively pollution-free environment in North America presented little need for public mask use after the Spanish Flu had passed. Further, the expected benefits of public mask use may also influence the frequency of mask wearing (van der Westhuizen et al., 2020). The distinctiveness of mask-use practice across cultures in recent history leads to the present research.

### Mask Use During the COVID-19 Pandemic

When COVID-19 erupted in their respective countries (e.g., China, India, Japan, and Vietnam), a majority of East Asians began to voluntarily wear masks for prevention purposes (Rab et al., 2020; Howard, 2021). In North America, opinions around public mask use were less uniform, with East Asian residents endorsing public mask use to a greater extent than their non-East Asian counterparts (He et al., 2020; Javid et al., 2020). Non-East Asian North Americans were relatively doubtful of the benefits of mask wearing as a preventative measure against COVID-19 (Couto, 2020). In part this doubt arose because of the mixed messages communicated by health authorities, which initially discouraged mask use by healthy individuals and understated the preventative efficacy of masks against COVID-19 outside of medical settings (Zhang et al., 2021). As the research evidence accumulated, some continued to exhibit ambivalence toward public mask use, despite efforts from health officials to promote mask wearing (Hensley, 2020).

The interethnic differences in mask use may have consequences for interethnic relations and public health within North America. Specifically, different cultural practices can lead to negative perceptions of public mask wearers in North America, especially wearers of East Asian descent. Since the onset of COVID-19, empirical research has documented the victimization of Asian Canadian and American mask wearers by those who were frustrated by the COVID-19 outbreaks (He et al., 2020). In particular, masks were linked to a threatening social identity that associated mask wearers with sources of disease contagion, flagging illness and threats to public health (Olivera-La Rosa et al., 2020; Rong, 2020). Especially early in the pandemic, the association between mask use and infectious diseases in North America instigated fear among East Asian residents, who grew concerned that they would be misjudged as contagious or carriers of COVID-19 if they wore masks in public areas (Cheung et al., 2020). To avoid “mask prejudice,” North Americans of East Asian descent even reported avoiding mask use despite a perceived need of respiratory protection (Pak, 2020), which may have serious public health consequences.

## Context and Objectives of the Current Studies

Like many other countries around the world, Canada has experienced substantial disruption from the COVID-19 pandemic. By 2022, over 2,800,000 cases of COVID-19 had been recorded, with a death toll exceeding 32,000 (Government of Canada, 2021b). The present research examined face mask use in Canada during the first wave of the COVID-19 pandemic (Study 1: April–May, 2020; Study 2: June, 2020). During this time period, medical masks were in short supply; WHO and the Canadian government recommended the use of masks in public; and the first governmental mandates for facemask use on public transportation were introduced. Although the majority of Canadians have received two doses of the COVID-19 vaccine by 2021 (Government of Canada, 2021c), the rates of infection still vary widely across provinces and regions within provinces. As a result, many Canadians still currently use diverse infection prevention measures, including mask-use in public areas.

While a substantial majority of Canadians supported public mask use since early in the pandemic (Berthiaume, 2020), resistance and doubts arose, especially among non-East Asian Canadians. On account of the mandates, a vocal minority demonstrated opposition to public mask use *via* anti-mask rallies (Bogart, 2020). The differential support for public mask use may have induced a sense of uneasiness among the ethnocultural groups who wore masks for respiratory protection. Such uneasiness may be especially compounded among Chinese Canadians, who were fearful of encountering “mask prejudice” due to their ethnic connection to China, where the first COVID-19 outbreak was located (Cheung et al., 2020).

Although many factors can account for national and ethnocultural differences in mask use, including past experiences of epidemics, the perceived threat of COVID, and social norms around mask use, relatively little comparative research has examined people’s beliefs and attitudes to public mask use, and the associations with mask-wearing. Conceptually, an attitude encompasses our affect, cognitions, and behaviors that together reflect an evaluation of a person, event, or issue (Bizer et al., 2003). In this research, we compare how subjective thoughts and feelings toward public mask use helps to achieve a clearer understanding of the rationales for mask-wearing among different ethnocultural groups, and how these might be differentially associated with public mask use.

### Research Objectives and Design

This research employed a mixed-methods design (Creswell and Plano Clark, 2011) to achieve a comparative understanding of the mask use attitudes and practice of Chinese and non-Chinese Canadians. Qualitative information was gleaned from group interviews that explored Chinese and non-East Asian Canadians’ perspectives and feelings about public mask use (Study 1), while quantitative data from an online survey examined the mask use frequency in the two groups, as well as their reasons for mask wearing prior to and during the COVID-19 pandemic (Study 2). We expect the qualitative and quantitative methods to complement one another in jointly examining the similarities and differences between Chinese and non-East Asian Canadians’ thoughts, feelings, and behaviors regarding public mask use. The findings are first discussed separately for each study, before they are integrated for interpretation in a general discussion. Both studies were approved by the Research Ethics Office at University of Alberta.

## Study 1

### Method

#### Participants

Twenty-six Chinese Canadians (*M*_*age*_ = 41.33 years, *SD* = 15.28; 15 females, 11 males), and 40 non-East Asian Canadians (*M*_*age*_ = 40.23 years, *SD* = 14.02; 25 females, 15 males) participated in online group interviews during April to May, 2020. The Chinese participants were recruited from an existing subject pool of Chinese immigrants. Their average length of residence in Canada was 11.81 years, *SD* = 7. Almost all participants had previously worn a face mask.

The non-East Asian Canadians were recruited from social media platforms. Twenty-seven of them were born in Canada, three were born in the United States, three in Iran, and the remaining participants were born in England, Italy, Mexico, Romania, Slovakia, United Kingdom, and Venezuela. Twenty-seven of the participants resided in the province of Alberta, nine in Ontario, two in British Columbia, two in Nova Scotia, one in Manitoba, and one in Saskatchewan. The average length of residence in Canada among the foreign-born participants was 21.37 years, *SD* = 15.38. At the time of the interviews, 57.4% of the participants had previously worn a mask in public.

#### Materials and Procedures

As a part of a bigger study that investigated Canadians’ attitudes and opinions of personal protective equipment during the first wave of the COVID-19 pandemic, participants responded to an online recruitment survey that collected informed consent, demographic information and previous use of masks. They were then invited to join group interviews that were conducted *via* Zoom by the first or third author, following a semi-structured script of prompts. The prompts that are relevant to the present study include, “Recalling your earliest experience of seeing some people wearing face masks in public, how did you react and why,” and “Why did you think people were wearing face masks?” Each interview consisted of three to six participants, and lasted between 60 and 90 min. Diverse perspectives were represented in each interview: each cohort varied in terms of age, country of birth, length and city of residence, and previous use of masks. The only exception was sex. Two same-sex groups (one all-female, one all-male) were created to ensure that comments and concerns which might be inhibited in mixed-group discussions could be documented. This group design was discontinued in subsequent interviews, as little additional information was obtained from the same-sex groups.

Each interview was video-recorded and transcribed using Zoom’s transcription service, with speech-to-text transcription verification by research assistants who audited the original interviews, took hand-written notes, and corrected the audio transcripts. The participants were given the opportunity to review and correct their statements on the transcripts before the data were analyzed, to ensure accuracy of the responses. The audio transcripts and hand-written notes from previous interviews were reviewed by the researchers before conducting the next interview, which facilitated the identification of new themes in the subsequent sessions. By the point of data saturation (Saunders et al., 2018), there were a total of five groups among the Chinese participants, and nine groups among the non-East Asian participants.

#### Analysis

The transcribed responses were imported into NVivo for thematic analysis, using an open-coding approach to capitalize on the exploratory nature of this study (Green, 2006). Three independent researchers coded the data. The coding process entailed three steps. First, all responses were thoroughly read to increase the researchers’ familiarization with the data. Second, preliminary codes were generated and assigned to each statement to capture its essence. In the third stage, all codes were intensively reviewed and collated based on shared similarities to form specific themes. Consistent with the study’s objective, the researchers attended to statements about the participants’ attitudes and beliefs about public mask use, while remaining open to other possible themes. Each statement could be coded with multiple themes. Discrepancies in coding and the development of themes were discussed among the coding team until consensus was reached. A summary of the final themes are presented in Table 1.

**TABLE 1:** Themes in Canadians’ reported attitudes toward mask wearers.

Chinese Canadians (*n* = 16)		Non-East Asian Canadians (*n* = 27)	
Themes	Number of participants	Themes	Number of participants
*Favorable attitudes*		*Favorable attitudes*	
1. Protection for oneself and others	5	1. Protection for oneself and others	7
2. Protection and safety	5	2. Respectful and responsible	4
3. Thankfulness	3	3. Cautious and serious about the pandemic	3
4. Respect	2	4. Comfort and safety	2
5. Approval	2	5. Appreciation	2
6. Hopefulness	1	6. Intelligence	1
*Other*		*Unfavorable attitudes*	
1. Mask-use as normal practice	2	1. Sick or ill	8
2. Relation to Chinese	1	2. “N95 Shaming”	4
		3. Strange	3
		4. Overreaction	2
		*Changing attitudes*	
		1. Growing acceptance and support	10
		2. Increase in positive perceptions	7
		*Other*	8
		1. Maintaining distance	5
		2. Asian	3

*The sample for each group only includes participants who had disclosed their opinions on the topics of interest during the interviews.*

### Results and Discussion

#### Chinese Canadians’ Attitudes Toward Mask Wearers

The results of the focus groups show that Chinese Canadians’ perceptions of mask wearers were more uniform than that of non-East Asian Canadians. Almost all of the Chinese participants (*n* = 14) held favorable attitudes toward mask wearers in their community. Specifically, the Chinese participants believed that mask wearers were protecting their own safety and that of others around them (*Protection for Oneself and Other*s; e.g., “I think wearing masks is… a form of protection for oneself. [and] a way of protecting others as well”), and the participants reported feeling protected and safe around mask wearers in public areas (*Protection and Safety*; e.g., “When I see more people wearing masks, I feel safer”). The participants were also thankful to mask wearers (*Thankfulness*; e.g., “I feel that finally people have this awareness [of wearing masks]… I am deeply touched, and thankful”), and disclosed *approval* and *respect* for people who wore masks in public areas (e.g., “I think they are doing a right thing. They should wear [masks],” “I will respect them”). One participant was hopeful of overcoming the COVID-19 pandemic as public mask use increased (*Hopefulness*; e.g., “As more people wear masks… I feel more confident that this pandemic will be taken under control”).

A few responses were grouped under the *Other* category, such that two participants regarded public mask use as normal, and hence did not have specific opinions (*Mask Use as Normal Practice*; e.g., “I feel that [mask-wearing] is pretty normal”). One participant associated public use of masks with having Chinese connections, either by ethnic origin or through having friends and/or significant others of Chinese descent (*Relation to Chinese*; e.g., “When I see a foreigner wear masks, I would wonder if he/she has a Chinese friend”). Such findings are consistent with previous literature that described public mask use as a widely endorsed practice in most East Asian communities worldwide (Chan, 2020).

#### Non-East Asian Canadians’ Attitudes Toward Mask Wearers

Compared to the Chinese Canadians, non-East Asian Canadians’ perceptions of mask wearers were more varied. Approximately half of the participants (*n* = 14) perceived mask wearers favorably, believing that mask wearers were attending to the safety and well-being of themselves and others (*Protection for Oneself and Others*; e.g., “[T]hey’re just trying to protect me and themselves”), and perceived mask wearers to be *respectful and responsible* (e.g., “if I see someone who [wore a] mask on the street. I would just think that they’re responsible and accountable for the safety measures”).

Those who perceived mask wearers favorably also thought that mask wearers were exercising caution and were taking the pandemic seriously (*Cautious and Serious About the Pandemic*; e.g., “Well, they’re just being cautious,” “I see they have masks on. So people are taking [the pandemic] serious[ly]”). Hence, the participants reported feeling comfortable and safe around people who wore masks in public areas (*Comfort and Safety*; e.g., “I think that I do feel safer when I see more and more people wearing masks outside,” “[When] someone’s wearing a mask, I actually feel kind of more comfortable”), and were appreciative of them (*Appreciation*; e.g., “I am very appreciative of them”). One participant perceived mask wearers as intelligent in preventative health practices (*Intelligence*; e.g., “When I see people, all the people wearing a mask or doing something I always think, okay, they are smart”).

There were also some participants who held relatively unfavorable perceptions toward mask wearers (*n* = 14), by associating public mask use with having a disease (*Sick or Ill*; e.g., “Anyone that I saw with a mask, I just assumed in my brain like that they were sick”). The participants also expressed their disapproval of public use of N95 masks, believing medical-grade masks ought to be reserved for healthcare workers (*N-95 Shaming*; “I feel if I saw someone wearing an N95… I’d be like, hey, why have you got that? Where’d you get that from? Why didn’t you give it to your nurse friend?”). Other perceptions, such as *strange* (e.g., “I actually saw a guy with a big gas mask on and whatever so that was strange”) and *overreaction* (e.g., “I find [mask-wearing] a sign of like the fact that the person is overly protecting himself or herself”), also emerged from responses.

Such relatively negative reactions were notably absent from the Chinese Canadians, who more uniformly spoke about the constructive aspects of public mask use during the COVID-19 pandemic. The more ambivalent views toward mask wearers among non-East Asian Canadians might be associated with the previous patterns of mask use in North America, where masks were predominantly worn by individuals who were potentially contagious or in close contact with sources of contagion (e.g., in medical clinics, hospitals; O’Kane and Baldwin, 2020).

Despite this noticeable difference in views, the analysis also captured a shift in non-East Asian Canadians’ perceptions of mask wearing in this rapidly evolving pandemic. Precisely, we noted discussion of a *growing acceptance and support* of public mask wearing (*n* = 14; e.g., “It became kind of accepting to see, you know, the cloth mask or the surgical mask being used”). This shift in attitude possibly reflects the beginning of normalization of public mask use in the Canadian context, where such behavior might have been regarded as peculiar prior to the COVID-19 pandemic. As well, the participants noted a transition in their perceptions toward mask wearers, which has become increasingly positive over time (*Increase in Positive Perceptions*; “I saw this man who was wearing like a very big mask and then I think I reacted in a way that was more so negative just because I wasn’t educated about it at the time… I was like oh man that guy’s probably sick but as time kind of passes you start getting more information you really see that that person was probably looking out for the rest of us”). This change in view again reflects the normalization of public mask use in Canada, which has helped to abate some of the negative stereotypes against mask wearers.

A couple of other themes also emerged, such that eight participants perceived public use of masks as a signal to maintain interpersonal distance (*Maintaining Distance*; e.g., “When I see somebody with a mask on, I want to steer clear,” “I would back off, oooh”), and three participants perceived people who wore masks publicly to be *Asian* (e.g., “[W]hen I saw a few people wearing masks… I was like, yes, that person’s Asian. It’s just Asian people wear masks… when you’re sick to be polite”).

In sum, the findings from Study 1 reflect particularly favorable perceptions of mask wearers among the Chinese Canadians, and ambivalent, yet changing perceptions among the non-East Asian Canadian participants, such that their attitudes were becoming more positive.

## Study 2

Study 1 provided a comparison between a Canadian group that has adopted facemask use as a normative practice (both in terms of how extensively it is used within a group and how favorably it is uniformly evaluated by group members), and one that is in the early stages of developing norms around mask use. Study 2 extends Study 1 by surveying a broad sample of Chinese and non-Asian Canadians to further understand their attitudes toward facemasks and facemask use, and to determine whether and how their attitudes predict the frequency with which they use facemasks in public settings.

### Method

#### Participants and Procedure

In collaboration with a Canadian public opinion research institute (blinded reference), 567 Chinese Canadians and 1,265 European Canadians over the age of 18 were recruited from an online panel associated with the research institute to participate in a nation-wide online survey. In contrast with Study 1, the non-Asian Canadian sample was restricted to Canadians of European descent on account of the infrequent public mask use in most European countries prior to the COVID-19 pandemic (Face-off over face-masks, 2020).

Among the Chinese Canadians subsample (*M*_*age*_ = 42.25 years, *SD* = 14.43; 264 females, 298 males, 5 identified as another gender), 42.2% of the participants resided in British Columbia, 38.3% in Ontario, 11.3% in Alberta, 5.1% in Quebec, 1.4% in Manitoba, 0.9% in Saskatchewan, and 0.9% in the Atlantic region. Approximately 47% of the Chinese Canadians were born in Canada. About 8.3% of the participants worked in healthcare, 24.2% worked in essential services (defined according to the Government of Canada’s classification; Public Safety Canada, 2021), and 42.7% of the participants identified as non-essential service workers.

For the European Canadians subsample (*M*_*age*_ = 49.5 years, *SD* = 16.38; 606 females, 616 males, 43 identified as another gender), 27.8% of the participants resided in Ontario, 22.7% in Quebec, 14.2% in Alberta, 12.3% in British Columbia, 11.6% in the Atlantic region, 5.8% in Manitoba, and 5.5% in Saskatchewan. About 92% of the European Canadians were born in Canada. Approximately 5% of the participants worked in healthcare, 27.2% worked in essential services, and 26.7% indicated as non-essential service workers.

The online survey took place between date and date, 2020, at a time when… (Zhang et al., 2021). All participants provided consent and received a small monetary incentive for completing the questionnaire.

#### Materials

The questionnaire assessed, among other issues, their frequency of and reasons for mask use prior to and during the COVID-19 pandemic.

##### Frequency of Mask Use

To assess the frequency of mask use prior to and during the COVID-19 pandemic, participants responded to questions that asked, “*Before the COVID-19 pandemic (that is, before there was any awareness of this coronavirus), how frequently, if ever, did you wear a face mask outside the home*,” and “*Now, during the COVID-19 pandemic, focusing on the past few weeks, how frequently, if at all, have you worn a face mask outside the home? If you have not left your home, please indicate that*.” Responses were collected on a Likert scale that ranged from 1 (*Never*) to 6 (*Almost Always*). An additional option was added to assess mask use during the pandemic, which clarifies, “I have not been outside in the past few weeks.”

##### Reasons for Mask Use

Two questions were devised to acquire Canadians’ reasons for mask use before and during the COVID-19 pandemic, which asked, “*Before/During the COVID-19 pandemic, what were your main reasons for wearing a face mask? Select all reasons that apply*.” A diverse range of reasons were articulated, based on a review of relevant literature that included Chinese and Canadian popular culture, news, and academic articles (e.g., see Flaskerud, 2020; O’Kane and Baldwin, 2020; Sun et al., 2020), in conjunction with the results from Study 1 and the multicultural research team’s subjective experiences in public mask use. Specifically, the reasons include, “*Prevent me from spreading disease/germs to others*,” “*Protect me from others’ diseases and germs*,” “*Give myself privacy, avoid being recognized*,” “*Because of pollen, smoke, or pollution in the air*,” “*To be fashionable*,” “*Because the weather was cold*,” “*Because friends/family wear facemasks*,” “*To publicly demonstrate social responsibility*,” and “*Required at work*.” Two additional reasons were incorporated to explain mask use during the COVID-19 pandemic, which are, “*To remind others to take the pandemic seriously*,” and “*Suggested by public health officials*.” Selected reasons were coded as “yes” responses, and unselected reasons were coded as “no” responses.

##### Perceived Risk of Death

Perceived risk was assessed with a single item that asked, “*Given that a person is infected with COVID-19, rate the risk that s/he will die as a result*.” Responses ranged from 1 (*No risk at all*) to 7 (*Very high risk*).

##### Covariates

Demographic information was also collected as control variables, including the participants’ age, gender, country of birth (Canadian-born vs. foreign-born), and income, which could presumably influence their mask wearing behaviors (see Haischer et al., 2020).

#### Analysis

We conducted a two-way within-subjects ANOVA to examine whether the frequency of mask use significantly differs before and during the COVID-19 pandemic among the Chinese and European Canadian participants. Next, we ran Cochran’s *Q* tests on the reasons for mask use before and during the pandemic to examine whether there were significant differences in the reasons of use for each group. McNemar’s *post hoc* tests were then used to locate the within-group differences and determine the ranked prevalence of the reasons. Further, Pearson’s Chi-square test was performed to compare between-group differences in the endorsement of each mask use reason.

To examine how each reason uniquely contributes to the frequency of public mask use, we performed several hierarchical multiple regression analyses using SPSS. Demographic variables, such as age, gender, education, income, and country of birth, were first entered into the equation, before inserting the specific mask use reasons. The perceived risk of death and pre-pandemic mask use frequency were entered as additional covariates in predicting the frequency of mask use during the COVID-19 pandemic. Both statistical significance (*p-*values ≤ 0.05) and practical significance (effect sizes > 0.10; e.g., partial η^2^, Cramer’s *V*, Pearson’s *r*) were considered when interpreting the significance of the results (Rosen and DeMaria, 2012).

### Results

#### (Pre-)pandemic Mask Use Frequency

**FIGURE 1:**
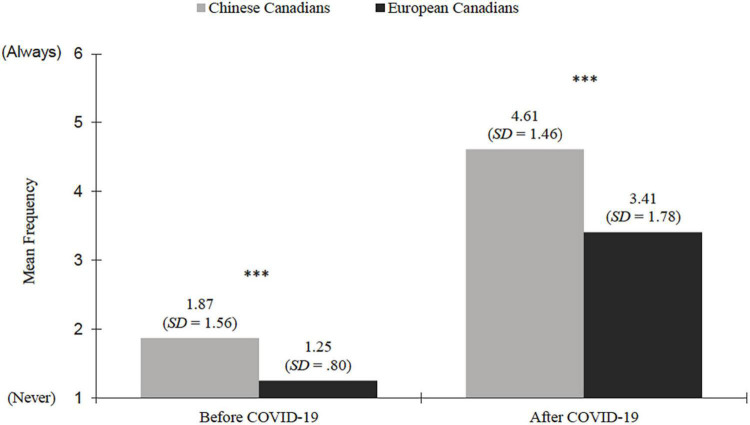
Frequency of mask use among Chinese Canadians (*n* = 549) and European Canadians (*n* = 1242) before and during the COVID-19 pandemic. *SD*, Standard Deviation, ^***^p < 0.001.

The frequency of mask use before and during the COVID-19 pandemic is displayed in Figure 1. We found a large main effect of time, *F*(1,1,789) = 2,587.99, *p* < 0.001, η^2^ = 0.59, such that both the Chinese and European Canadians reported more frequent use of masks during the COVID-19 pandemic compared to before. In general, Chinese Canadians tend to wear masks more often than European Canadians, *F*(1,1,789) = 274.97, *p* < 0.001, η^2^ = 0.13. A very small but statistically significant interaction effect between ethnicity and time also emerged, *F*(1,1,789) = 36.31, *p* < 0.001, η^2^ = 0.02, which indicated that Chinese Canadians were slightly more likely than European Canadians to wear a mask during the COVID-19 pandemic compared to before. Overall, these findings reflect a more prevalent use of masks among Chinese Canadians prior to and during the first wave of the pandemic.

#### Reasons for Mask Use

**FIGURE 2:**
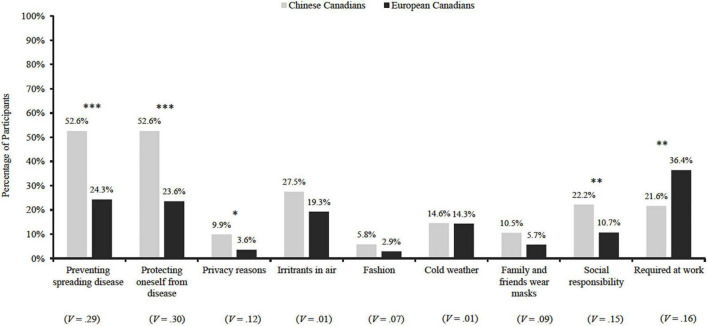
Mask-wearing reasons among Chinese Canadians (*n* = 171) and European Canadians (*n* = 140) before the COVID-19 pandemic. * *p* < 0.05, ^**^p < 0.01, ^***^P < 0.001, *V*, Cramer’s *V*.

Cochran’s *Q* tests indicate that the reasons to wear a mask significantly differed for the Chinese Canadians prior to the pandemic, χ^2^(8) = 230.50, *p* < 0.001, and during the pandemic, χ^2^(10) = 2,041.75, *p* < 0.001 (see Figure 2). According to McNemar’s *post hoc* tests, the pre-pandemic mask use reasons ranked in the following order, beginning with the most prevalent reasons: (1) to prevent spreading diseases to others and to protect oneself from others’ diseases; (2) to block contact with irritants in air, to demonstrate social responsibility, and because it was required by work; (3) because the weather was cold, because my family and friends wear masks, and in order to give myself privacy, and 4) to be fashionable. During the pandemic, pandemic-related reasons (e.g., disease prevention, public health recommendations, social responsibility, and reminding others to take the pandemic seriously) took precedence over the rest.

Likewise, we noted significant differences in the reasons for mask use among the European Canadians both before the pandemic, χ^2^(8) = 102.35, *p* < 0.001, and during the pandemic, χ^2^(10) = 3516.24, *p* < 0.001. Specifically, before the pandemic, the reasons ranked as follows: (1) required at work, to prevent spreading diseases, and to protect oneself from diseases; (2) to block contact with irritants in air, because of cold weather; (3) to show social responsibility, and because family and friends wear masks; and (4) to be fashionable and to maintain privacy. Similar to the Chinese Canadians, health-related reasons for public mask use took precedence over the others during the COVID-19 pandemic.

For between-group comparisons, Chinese Canadians tend to wear masks for more diverse purposes than European Canadians do. Specifically, significantly more Chinese Canadians reported wearing a mask for the following reasons prior to the pandemic (see Figure 2), such as to prevent the spread of diseases to others, χ^2^(1,311) = 25.80, *p* < 0.001, *V* = 0.29, to protect oneself from others’ germs and diseases, χ^2^(1,311) = 27.19, *p* < 0.001, *V* = 0.30, to stay private in public areas, χ^2^(1,311) = 4.75, *p* = 0.03, *V* = 0.12, and to publicly demonstrate social responsibility, χ^2^(1,311) = 7.21, *p* < 0.01, *V* = 0.15. On the other hand, more European Canadians than Chinese Canadians wore masks due to work requirements, χ^2^(1,311) = 8.30, *p* < 0.01, *V* = 0.16.

**FIGURE 3:**
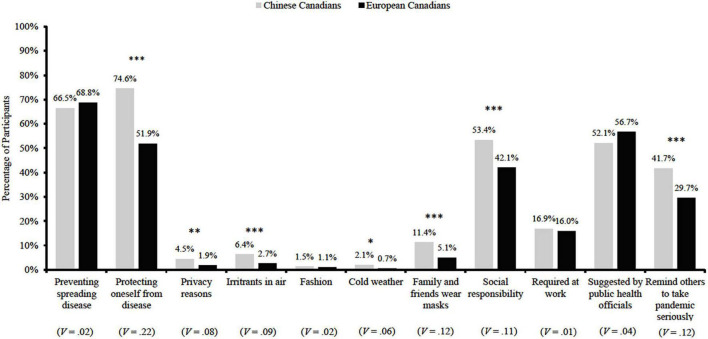
Mask-wearing reasons among Chinese Canadians (*n* = 528) and European Canadians (*n* = 965) during the COVID-19 pandemic. *P < 0.05, ^**^P < 0.01, ^***^p < 0.001, *V*, Cramer’s *V*.

During the pandemic, significantly more Chinese Canadians (vs. European Canadians) wore a mask to protect themselves from others’ germs and diseases, χ^2^(1,1,493) = 73.27, *p* < 0.001, *V* = 0.22, because their family and friends wore masks, χ^2^(1,1,493) = 19.93, *p* < 0.001, *V* = 0.12, to publicly demonstrate social responsibility, χ^2^(1,1,493) = 17.65, *p* < 0.001, *V* = 0.11, and to remind others to take the pandemic seriously, χ^2^(1,1,493) = 21.64, *p* < 0.001, *V* = 0.12 (see Figure 3).

These findings suggest two important messages. First, expectations around the preventive efficacy of masks initially differed between the Chinese and European Canadians, with significantly less European Canadians choosing to wear masks as a means of protection for *themselves*. This finding is consistent with the initial doubts Canadians reported around (1) the protective efficacy of masks, and (2) the benefits of masking as a means of self-protection for healthy individuals. In turn, this difference in the expectations regarding masks may impact the perceived importance of mask use during the pandemic, which is illustrated by significantly less number of European Canadians (vs. Chinese Canadians) who used masks to signal others to take the pandemic seriously. Second, consistent with current literature (e.g., see Mahbub et al., 2020; Lu et al., 2021), our results suggest a collectivistic attitude of mask use among the Chinese participants, such that in this critical time of the pandemic, significantly more Chinese participants reported wearing masks to demonstrate social responsibility, and because their family and friends were also doing so.

Overall, the results are in line with the literature we reviewed, which indicated that early in the pandemic masks tend to be used for various reasons in Chinese societies, including but not limited to the protection of self and others to preserve group well-being, whereas in North America, masks were predominately used as a personal protective gear in high-risk environments (e.g., construction sites and hospitals; O’Kane, 2020). Such diversified use of face masks among the Chinese population might reinforce both the descriptive (prevalence) and prescriptive norms (social acceptability) around public mask use, whereas the restricted use of masks in worksites among the European Canadians limited people’s acceptance of masks outside those specific contexts.

#### Predictors of Mask Use Frequency

Having established group differences in the frequency and reasons of public mask use, we conducted hierarchical multiple regression analyses whether particular reasons are linked to mask use, separately for Chinese and European Canadians.

##### Chinese Canadians

As shown in Table 2, neither the demographic variables nor the pre-pandemic mask use reasons significantly predicted Chinese Canadians’ mask wearing frequency before the COVID-19 pandemic. Birth country had a marginal effect on mask use, such that those who were born outside of Canada wore masks more often than their Canadian-born counterparts. This finding implies that either other unidentified reasons account for mask use, or mask use is such a taken-for-granted practice among the Chinese Canadians that it is simply a widespread, normative habit, that particular personal motives do not consistently predict the behavior. However, it is important to note that this arguably normative use may be weakened among Canadian-born Chinese, as they adapt into the Canadian society which endorsed public mask use to a lesser extent. Otherwise stated, acculturation, although not a prominent focus of this article, may exert influence over old cultural habits and modify one’s traditional practices.

**TABLE 2:** Hierarchical regression results for public mask use among Chinese Canadians before and during the COVID-19 pandemic.

			*B* [95% CI]	SE (*B*)	β	*r*	*sr* ^2^
Pre-pandemic mask use (*n* = 171)	Step 1						
	*R*^2^ = 0.04	Age	0.004 [−0.01, 0.02]	0.01	0.04	0.07	0.002
	Δ*R*^2^ = 0.04	Female	−0.15 [−0.63, 0.32]	0.24	−0.05	−0.02	0.002
		Other-gender	0.96 [−0.80, 2.71]	0.89	0.08	0.07	0.01
		Education	−0.08 [−0.25, 0.09]	0.08	−0.08	−0.06	0.01
		Income	−0.01 [−0.17, 0.15]	0.08	−0.01	−0.03	<0.001
		Birth country	0.54 [0.04, 1.04]	0.25	0.17*	0.15	0.02
	Step 2						
	*R*^2^ = 0.10	Age	0.01 [−0.01, 0.03]	0.01	0.09	0.07	0.01
	Δ*R*^2^ = 0.06	Female	−0.14 [−0.63, 0.34]	0.25	−0.05	−0.02	0.002
		Other-gender	1.07 [−0.76, 2.89]	0.92	0.09	0.07	0.01
		Education	−0.08 [−0.06, 0.09]	0.09	−0.07	−0.06	0.004
		Income	−0.01 [−0.17, 0.15]	0.08	−0.01	−0.03	<0.001
		Birth country	0.49 [−0.02, 0.97]	0.26	0.15	0.15	0.01
		Preventing spreading diseases	0.05 [−0.46, 0.55]	0.26	0.02	0.06	<0.001
		Protecting oneself from diseases	0.26 [−0.25, 0.78]	0.26	0.09	0.14	0.01
		Privacy reasons	0.07 [−0.77, 0.91]	0.43	0.01	0.05	<0.001
		Irritants in air	−0.21 [−0.75, 0.34]	0.27	−0.06	−0.09	0.003
		Fashion	0.16 [−0.87, 1.19]	0.52	0.03	0.01	<0.001
		Cold weather	0.14 [−0.55, 0.83]	0.35	0.03	0.01	0.001
		Family and friends wear masks	0.59 [−0.25, 1.44]	0.43	0.12	0.15*	0.01
		Social responsibility	0.36 [−0.23, 0.95]	0.30	0.10	0.16*	0.01
		Required at work	0.28 [−0.29, 0.85]	0.28	0.08	0.07	0.01
Pandemic mask use (*n* = 528)	Step 1						
	*R*^2^ = 0.05	Age	−0.004 [−0.01, 0.003]	0.004	−0.05	−0.03	0.002
	Δ*R*^2^ = 0.05***	Male	0.17 [−0.06, 0.39]	0.11	0.06	0.06	0.004
		Other-gender	0.56 [−0.70, 1.82]	0.64	0.04	0.02	0.001
		Education	−0.002 [−0.08, 0.08]	0.04	−0.002	0.02	<0.001
		Income	0.07 [0.003, 0.14]	0.04	0.09*	0.07	0.01
		**Birth country**	0.52 [0.30, 0.75]	0.11	0.20***	0.18***	0.04
	Step 2						
	*R*^2^ = 0.09	Age	−0.004 [−0.01, 0.004]	0.004	−0.04	−0.03	0.002
	Δ*R*^2^ = 0.04***	Male	0.16 [−0.06, 0.37]	0.11	0.06	0.06	0.004
		Other-gender	0.21 [−1.03, 1.45]	0.63	0.01	0.02	0.001
		Education	0.01 [−0.08, 0.09]	0.04	0.01	0.02	<0.001
		Income	0.08 [0.01, 0.15]	0.04	0.10*	0.07	0.01
		**Birth country**	0.42 [0.20, 0.64]	0.11	0.16***	0.18***	0.04
		**Pre-pandemic mask use**	0.17 [0.10, 0.23]	0.04	0.20***	0.23***	0.04
	Step 3						
	*R*^2^ = 0.2	Age	−0.004 [−0.01, 0.004]	0.004	−0.04	−0.03	0.002
	Δ*R*^2^ = 0.16***	Male	0.13 [−0.07, 0.33]	0.10	0.05	0.06	0.004
		Other-gender	0.15 [−1.01, 1.30]	0.58	0.01	0.02	0.001
		Education	−0.01 [−0.08, 0.07]	0.04	−0.01	0.02	<0.001
		Income	0.06 [0.001, 0.127]	0.03	0.08*	0.07	0.01
		**Birth country**	0.40 [0.19, 0.61]	0.11	0.15***	0.18***	0.04
		**Pre-pandemic mask use**	0.19 [0.12, 0.25]	0.03	0.23***	0.23***	0.04
		**Perceived risk**	0.13 [0.06, 0.20]	0.04	0.15***	0.22***	0.02
		**Preventing spreading diseases**	0.54 [0.31, 0.77]	0.12	0.20***	0.25***	0.03
		**Protecting oneself from diseases**	0.39 [0.15, 0.63]	0.12	0.13**	0.21***	0.02
		Privacy reasons	−0.03 [−0.53, 0.47]	0.25	−0.01	0.02	0.003
		Irritants in air	−0.13 [−0.57, 0.31]	0.22	−0.03	0.06	0.001
		Fashion	0.25 [−0.59, 1.09]	0.43	0.02	0.02	0.001
		Cold weather	−0.31 [−1.04, 0.43]	0.37	−0.03	−0.05	0.001
		Family and friends wear masks	−0.34 [−0.66, −0.01]	0.17	−0.08*	−0.05	0.01
		Social responsibility	0.14 [−0.10, 0.37]	0.12	0.05	0.16***	0.002
		Required by work	0.15 [−0.12, 0.42]	0.14	0.04	0.06	0.002
		Suggested by public health officials	0.12 [−0.10, 0.34]	0.11	0.05	0.10*	0.002
		**Remind others to take the pandemic seriously**	0.29 [0.04, 0.53]	0.13	0.11*	0.27***	0.01

*CI, confidence interval; SE, standard error; r, zero-order correlation; sr^2^, squared semi-partial correlation.*

Birth country: 0 = Canadian-born, 1 = Foreign-born. Due to high multicollinearity between males and females (tolerance <0.001), only one of the two gender groups are included in the models. No multicollinearity was detected among the predictors; tolerance scores >0.60; VIF scores <1.5. Bolded = significant predictors. *p < 0.05; **p < 0.01; ***p < 0.001.

In explaining Chinese Canadians’ mask use frequency during the COVID-19 pandemic, the demographic variables accounted for 5% of the total variance in the dependent variable, *F*(6,521) = 4.48, *p* < 0.001. Specifically, participants who were born outside of Canada and those with higher income tend to wear masks more frequently. Pre-pandemic mask use frequency explained an additional 4% of the total variance in pandemic mask use frequency, Δ*F*(1,520) = 21.89, *p* < 0.001.

In the final step, the specific reasons, along with the perceived risk of contracting COVID-19, explained an additional 16.3% of the total variance in pandemic mask-use frequency, Δ*F*(12,508) = 8.98, *p* < 0.001. Particularly, the participants wore masks more frequently when they perceived greater risk of death in contracting COVID-19 and wanted to protect themselves and others from respiratory infections, in addition to signaling others to take the pandemic seriously. These findings suggest that Chinese Canadians perceived public mask use as an important strategy in the battle against COVID-19. Perhaps due to having more experience and knowledge about mask-use in public disease prevention (Yang, 2014; Greenhalgh et al., 2020), Chinese Canadians indicated little influence from the recommendations of public health officials. Further, the findings also suggest the impact of social norms in facilitating certain health practices. For instance, foreign-born Chinese Canadians (who might have had greater personal contact with the “mask norms” in China) were more likely to continue doing so. Moreover, by wearing masks one could signal to others the importance of the practice, potentially changing social norms of mask-wearing in public.

##### European Canadians

The demographic variables accounted for 20% of the total variation in the mask use frequency of European Canadians before the COVID-19 pandemic (see Table 3), *F*(6,133) = 5.40, *p* < 0.001. Particularly, participants who identified other than male or female were significantly more likely to wear masks in public prior to the pandemic. Further, the reasons entered in Step 2 explained an additional 11% of the total variance in European Canadians’ frequency of pre-pandemic mask use, Δ*F*(9,124) = 2.17, *p* = 0.03. Among the reasons, significant predictors include demonstrating social responsibility, and wearing masks because one’s family and friends do so. The predictive effect of gender ceased when the other reasons were accounted for. These results suggest that since public mask use was less widely endorsed by European Canadians before the pandemic, they may draw on a sense of personal obligation and concordance with significant others (e.g., relying on prescriptive norms; Baumeister and Vohs, 2007) to rationalize and/or guide their actions.

**TABLE 3:** Hierarchical regression results for public mask use among European Canadians before and during the COVID-19 pandemic.

			*B* [95% CI]	SE (*B*)	β	*r*	*sr* ^2^
Pre-pandemic mask use	Step 1						
(*n* = 140)	*R*^2^ = 0.20	Age	0.01 [0, 0.03]	0.01	0.17*	0.04	0.02
	Δ*R*^2^ = 0.20***	Male	−0.04 [−0.44, 0.35]	0.20	−0.02	−0.20*	<0.001
		**Other-gender**	1.14 [0.66, 1.63]	0.25	0.43***	0.39***	0.13
		Education	−0.02 [−0.13, 0.08]	0.05	−0.04	0.01	0.001
		Income	−0.05 [−0.17, 0.07]	0.06	−0.06	−0.12	0.004
		Birth country	−0.41 [−1.08, 0.25]	0.34	−0.10	−0.11	0.01
	Step 2						
	*R*^2^ = 0.31	Age	0.01 [−0.004, 0.02]	0.01	0.11	0.04	0.01
	Δ*R*^2^ = 0.11*	Male	0.16 [−0.25, 0.56]	0.20	0.07	−0.20*	0.003
		**Other-gender**	1.05 [0.53, 1.56]	0.26	0.39***	0.39***	0.09
		Education	−0.01 [−0.12, 0.10]	0.05	−0.02	0.01	<0.001
		Income	−0.04 [−0.16, 0.08]	0.06	−0.06	−0.12	0.003
		Birth country	−0.53 [−1.22, 0.15]	0.34	−0.12	−0.11	0.01
		Preventing spreading diseases	0.07 [−0.38, 0.53]	0.23	0.03	0.07	0.001
		Protecting oneself from diseases	0.39 [−0.07, 0.84]	0.23	0.15	0.09	0.02
		Privacy reasons	0.52 [−0.47, 1.51]	0.50	0.09	0.01	0.01
		Irritants in air	0.18 [−0.30, 0.65]	0.24	0.06	−0.05	0.003
		Fashion	0.03 [−1.03, 1.09]	0.53	0.004	<0.001	<0.001
		Cold weather	−0.39 [−0.89, 0.11]	0.25	−0.12	−0.17	0.01
		**Family and friends wear masks**	0.82 [0.02, 1.61]	0.40	0.17*	0.22**	0.02
		**Social responsibility**	0.96 [0.35, 1.57]	0.31	0.27**	0.32***	0.05
		Required at work	0.18 [−0.26, 0.62]	0.22	0.08	−0.17*	0.003
Pandemic mask use	Step 1						
(*n* = 965)	*R*^2^ = 0.08	**Age**	0.02 [0.01, 0.02]	0.003	0.20***	0.20***	0.04
	Δ*R*^2^ = 0.08***	**Male**	−0.33 [−0.50, −0.15]	0.09	−0.12***	−0.11***	0.01
		Other-gender	−0.44 [−0.88, 0.001]	0.23	−0.06*	−0.06	0.004
		**Education**	0.11 [0.06, 0.16]	0.03	0.13***	0.14**	0.02
		Income	0.01 [−0.06, 0.05]	0.03	−0.01	0.04	<0.001
		Birth country	0.21 [−0.10, 0.52]	0.16	0.04	0.06	0.002
	Step 2						
	*R*^2^ = 0.08	**Age**	0.02 [0.01, 0.02]	0.003	0.20***	0.20***	0.04
	Δ*R*^2^ = 0.004	**Male**	−0.33 [−0.50, −0.15]	0.09	−0.12***	−0.11***	0.01
		Other-gender	−0.68 [−1.18, −0.18]	0.26	−0.10***	−0.06	0.004
		**Education**	0.11 [0.06, 0.16]	0.03	0.13***	0.14***	0.02
		Income	0.001 [−0.06, 0.06]	0.03	−0.001	0.04	<0.001
		Birth country	0.22 [−0.09, 0.53]	0.16	0.04	0.06	0.002
		Pre-pandemic mask use	0.11 [−0.002, 0.22]	0.06	0.07	0.02	0.004
	Step 3						
	*R*^2^ = 0.36	**Age**	0.01 [0.01, 0.02]	0.002	0.12***	0.20***	0.04
	Δ*R*^2^ = 0.28***	Male	−0.14 [−0.30, 0.01]	0.08	−0.05	−0.11***	0.01
		Other-gender	0.03 [−0.41, 0.47]	0.22	0.004	−0.06	0.004
		**Education**	0.06 [0.01, 0.10]	0.02	0.07*	0.14***	0.02
		Income	−0.001 [−0.05, 0.04]	0.02	−0.001	0.04	<0.001
		Birth country	0.16 [−0.10, 0.42]	0.13	0.03	0.06	0.002
		Pre-pandemic mask use	0.16 [0.06, 0.25]	0.05	0.10**	0.02	0.004
		**Perceived risk**	0.15 [0.09, 0.22]	0.03	0.13***	0.27***	0.01
		**Preventing spreading diseases**	0.68 [0.51, 0.86]	0.09	0.23***	0.41***	0.04
		**Protecting oneself from diseases**	0.49 [0.33, 0.65]	0.08	0.18***	0.35***	0.03
		Privacy reasons	0.12 [−0.42, 0.66]	0.28	0.01	0.03	<0.001
		Irritants in air	0.27 [−0.18, 0.72]	0.23	0.03	0.05	0.001
		Fashion	−0.02 [−0.72, 0.68]	0.36	−0.001	−0.02	<0.001
		Cold weather	−0.33 [−1.19, 0.54]	0.44	−0.02	−0.05	<0.001
		Family and friends wear masks	−0.09 [−0.42, 0.25]	0.17	−0.01	0.03	<0.001
		**Social responsibility**	0.31 [0.13, 0.49]	0.09	0.11**	0.35***	0.01
		Required by work	0.11 [−0.09, 0.32]	0.10	0.03	−0.09**	0.001
		**Suggested by public health officials**	0.33 [0.16, 0.49]	0.08	0.12***	0.32***	0.01
		**Remind others to take the pandemic seriously**	0.44 [0.25, 0.63]	0.10	0.14***	0.37***	0.01

*CI, confidence interval; SE, standard error; r, zero-order correlation; sr^2^, squared semi-partial correlation.*

Birth country: 0 = Canadian-born, 1 = Foreign-born. Due to high multicollinearity between males and females (tolerance <0.001), only one of the two gender groups are included in the models. No multicollinearity was detected among the predictors; tolerance scores ≥0.60; VIF scores <1.80. Bolded = significant predictors. *p ≤ 0.05; **p < 0.01; ***p < 0.001.

During the COVID-19 pandemic, the demographic variables contributed significantly to the model, *F*(6,958) = 13.24, *p* < 0.001, and accounted for 7.7% of the total variance in the mask use frequency of the European Canadians. Specifically, participants with older age and higher education tend to wear masks more frequently in the pandemic. A gender variation was also found, such that males were significantly less likely to wear masks than females. Incorporating pre-pandemic mask use frequency in the second stage created little incremental change to the model, which explained an additional 0.4% of the total variance in non-East Asian Canadians’ mask-use frequency during the pandemic, Δ*F*(1,957) = 3.75, *p* = 0.05. In the last stage, the specific reasons and perceived risk of death explained another additional 28% of the variance in non-East Asian Canadians’ pandemic mask use frequency, Δ*F*(12,945) = 34.14, *p* < 0.001. Specifically, perceived risk of death, along with reasons such as protecting oneself and others from respiratory diseases, demonstrating social responsibility, following the suggestions of public health officials, and reminding others to take the pandemic seriously were all significant predictors of more frequent mask use during the COVID-19 pandemic. Although gender ceased as a significant predictor when the other reasons were accounted for, age and education remained significant, which possibly speaks to the impact of physical vulnerabilities with older age and access to scientific information, respectively.

## General Discussion

The main purpose of this research was to compare attitudes toward mask wearers as well as reasons for wearing facemasks across Chinese and non-East Asian Canadians before and during the COVID-19 pandemic, and to determine whether risk perceptions and reasons differentially predicted facemask use in these two groups. Study 1 provided an in-depth exploration of Chinese and non-East Asian Canadians’ perceptions of mask wearers. Study 2 examined the differential association between reasons for wearing a facemask and the frequency of public mask use across the two ethnocultural groups. In these two studies, we observed a prevalent and diversified use of face masks among Chinese Canadians prior to and during the COVID-19 pandemic. The frequent use of masks may be reinforced by the relatively favorable attitudes Chinese Canadians held toward public mask wearing, such as perceiving mask wearers to be respectful and responsible. As Chinese Canadians may already be knowledgeable about the benefits and practice of public mask use, their mask wearing practice relied less on public health recommendations. This possibility is consistent with the early sightings of Asian mask wearers in public settings in North America (He et al., 2020), which occurred months before masks were widely recommended and mandated for use in the pandemic.

In contrast, the early mask use hesitancy among non-East Asian Canadians’ might be associated with their ambivalent attitudes toward public mask use. Specifically, although some non-East Asian Canadians perceived mask wearers to be socially responsible, others perceived mask wearers to be ill, strange, and overreacting. Potentially due to such negative reactions, non-East Asian Canadians tend to wear masks publicly much less often, and may be more judgmental and hostile toward mask wearers earlier in the pandemic (Rong, 2020).

Despite the initially negative reactions, non-East Asian Canadians’ attitudes toward public mask use shifted significantly during the pandemic to become increasingly positive. It might be that as Canadians have grown accustomed to mask wearing in communities, regardless of current health conditions, it helps to eradicate the initial negative stereotypes that associated public mask use with the presence of an illness (Klitzman, 2020). As one’s knowledge and understanding about masks continues to grow, stigmatization toward mask wearers and “mask-prejudice” may decrease correspondingly (and vice versa).

### Implications for Public Health Strategies

The present studies have identified several ethnocultural differences in attitudes toward public mask use, which underscore a need to divert public health strategies to meet each ethnocultural group “where they are” to achieve maximal protection. For instance, given that Chinese individuals are generally more knowledgeable and experienced with mask use (Yang, 2014), information on the preventative efficacy of masks against diseases might be of little value to this group in a public health crisis. However, a more supportive and stigma-free mask use environment may help to reinforce and preserve their mask wearing in public areas. This might be achieved with an information campaign that targets on eradicating misconceptions associated with mask wearing, as well as clarifying its public health significance during the pandemic to promote positive mask attitudes and greater use (Lai et al., 2021; Zhang et al., 2021).

On the other hand, since public mask use was more ambivalent among non-East Asian Canadians (e.g., European Canadians), this population may require more “active information” to initiate an attitudinal change. Such information might include public health recommendations and scientific evidence that clearly explains the rationale behind the recommended measures (Zhang et al., 2021). Moreover, ensuring this information is readily accessible to the public may be especially valuable to those who do not have convenient access to scientific information. Further, the age differences in mask use call for targeted messaging and behavioral modification strategies to increase mask wearing among the younger population. Strategies might include the use of social media influencers to connect masks with a positive social identity, appealing to personal values, and addressing emotional challenges that prevent mask use (Hutchins et al., 2020).

Beyond providing education on the scientific benefits of mask use, social and personal reinforcers could also be utilized to increase the adoption of public mask wearing. Social norms are a powerful force in shaping health-related behaviors (Ball et al., 2010). Particularly, the development of prescriptive norms for public mask use, such as increased use among one’s family, friends, neighbors, and colleagues, may induce the pressure for one to conform to avoid social disapproval. Research has also shown that in collectivistic societies, a sense of collective and civic responsibility was associated with more frequent mask use (Lu et al., 2021). In more individualistic societies where social responsibility is less prominent, personal appeals may be of value (Higgins et al., 2012). These may include stressing the link between public mask use and the longed future of life returning to “normal,” where people could freely enjoy the events and amenities which have been temporarily restricted or suspended because of the pandemic. In sum, a clearer understanding of the severity of the pandemic and the expected benefits of public mask use, coupled with facilitative social norms could all contribute to a greater likelihood of public mask use among the non-East Asian population.

### Limitations and Future Research Recommendations

There are a few limitations that point to future research directions. First, the current research utilizes cross-sectional studies to explore how subjective perceptions and affect may impact mask use behaviors, which does not allow one to draw causal inferences from the findings. To assess the predictive effects of subjective attitudes on health-related behaviors, longitudinal research methods may be a better alternative to examine causal effects over time. Second, it is important to note that our findings reflect the subjective attitudes of Chinese and non-East Asian Canadians on public mask use relatively early in the pandemic. Given the rapidly evolving circumstances, follow-up studies should compare current attitudes and practices on public mask wearing with those of a year ago. Third, to understand how preventative behavioral norms become established, it might be helpful to examine people’s attitudes toward public mask use after the mask mandates lift, especially among ethnic groups for whom mask wearing was less prevalent. Such investigation would help to determine whether current public mask use norms would persist after the pandemic fades, and shed light on the contributing factors that either help to maintain or alter health-related behavioral norms.

## Conclusion

Facemasks are a critical safety tool in the COVID-19 pandemic (Green et al., 2020). Despite abundant literature citing the efficacy of masks in the prevention of COVID-19 (e.g., see Kaslow et al., 2020; Howard et al., 2021; Li et al., 2021), mask use notably differed between Chinese and non-Chinese Canadians in the beginning of the pandemic. Using a mixed-methods approach, the present studies showed significant differences in the attitudes, reasons, and frequency of public mask use between Chinese and non-East Asian Canadians prior to and during the COVID-19 pandemic. The findings also showed that, not only did the groups differ in terms of which reasons justified their facemask use, but also for both groups, different clusters of reasons predicted their facemask use before and during the pandemic. Such differences present a need to consider between-group differences in promoting public health measures to achieve optimal effectiveness. Pertaining to public mask use, a clearer understanding of the facilitators and barriers to use that are unique to different ethnocultural groups help to foster intergroup understanding, and assist public health authorities to adjust their promotional strategies accordingly to achieve greater public influence. These implications are not only pertinent to the current pandemic, they may also extend to other health emergencies to safeguard public well-being.

## Data Availability Statement

The datasets presented in this study can be found in online repositories. The names of the repository/repositories and accession number(s) can be found below: https://doi.org/10.7939/r3-yz5x-e253.

## Ethics Statement

The studies involving human participants were reviewed and approved by the Research Ethics Office – University of Alberta. The participants provided their written informed consent to participate in this study.

## Author Contributions

All authors contributed to the design of the two studies in this manuscript. YZ and HY-L conducted the qualitative interviews. YZ conducted the data analyses and wrote the manuscript with the assistance of the KN. All authors provided feedback to the manuscript prior to its submission.

## Conflict of Interest

The authors declare that the research was conducted in the absence of any commercial or financial relationships that could be construed as a potential conflict of interest.

## Publisher’s Note

All claims expressed in this article are solely those of the authors and do not necessarily represent those of their affiliated organizations, or those of the publisher, the editors and the reviewers. Any product that may be evaluated in this article, or claim that may be made by its manufacturer, is not guaranteed or endorsed by the publisher.
